# Preservation of Myocardial Perfusion and Function by Keeping Hypertrophied Heart Empty and Beating for Valve Surgery: An In Vivo MR Study of Pig Hearts

**DOI:** 10.1155/2017/4107587

**Published:** 2017-03-20

**Authors:** Jian Wang, Bo Xiang, Jixian Deng, Hung-Yu Lin, Darren H. Freed, Rakesh C. Arora, Ganghong Tian

**Affiliations:** ^1^Department of Vascular Surgery, Union Hospital, Tongji Medical College, Huazhong University of Science and Technology, 1277 Jiefang Avenue, Wuhan 430022, China; ^2^National Research Council of Canada, 435 Ellice Avenue, Winnipeg, MB, Canada R3B 1Y6; ^3^Department of Physiology, Faculty of Medicine, University of Manitoba, 727 McDermot Avenue, Winnipeg, MB, Canada R3E 3P5; ^4^Department of Pharmacology and Therapeutics, Faculty of Medicine, University of Manitoba, 753 McDermot Avenue, Winnipeg, MB, Canada R3E 0T6; ^5^Division of Cardiac Surgery, University of Alberta Hospital, 8440-112 Street, Edmonton, AB, Canada T6G 2B7; ^6^Cardiac Science Program, Institute of Cardiovascular Science, St. Boniface General Hospital, 409 Tache Avenue, Winnipeg, MB, Canada R2H 2A6

## Abstract

*Objectives*. Normothermic hyperkalemic cardioplegia arrest (NHCA) may not effectively preserve hypertrophied myocardium during open-heart surgery. Normothermic normokalemic beating perfusion (NNBP), keeping hearts empty-beating, was utilized as an alternative to evaluate its cardioprotective role.* Materials and Methods*. Twelve hypertrophied pig hearts at 58.6 ± 7.2 days after ascending aorta banding underwent NNBP and NHCA, respectively. Near infrared myocardial perfusion imaging with indocyanine green (ICG) was conducted to assess myocardial perfusion. Left ventricular (LV) contractile function was assessed by cine MRI. TUNEL staining and western blotting for caspase-3 cleavage and cardiac troponin I (cTnI) degradation were conducted in LV tissue samples.* Results*. Ascending aortic diameter was reduced by 52.7% ± 0.4% at approximately fifty-eight days after banding. LV wall thickness was significantly higher in aorta banding than in sham operation. Myocardial blood flow reflected by maximum ICG absorbance value was markedly higher in NNBP than in NHCA. The amount of apoptotic cardiomyocyte was significantly lower in NNBP than in NHCA. NNBP alleviated caspase-3 cleavage and cTnI degradation associated with NHCA. NNBP displayed a substantially increased postoperative ejection fraction relative to NHCA.* Conclusions*. NNBP was better than NHCA in enhancing myocardial perfusion, inhibiting cardiomyocyte apoptosis, and preserving LV contractile function for hypertrophied hearts.

## 1. Introduction

Conventional cardioplegia confers sufficient myocardial protection for most patients with preserved ventricular function and leads to a complete postoperative recovery of cardiac function. In patients with compromised cardiac function, such as those with severe myocardial hypertrophy, conventional cardioplegia does not provide adequate myocardial preservation [1, 2]. This is because the hypertrophied hearts are more vulnerable to the detrimental effects of cardioplegia, such as coronary endothelial dysfunction, cardiomyocyte apoptosis, and myocardial stunning.

Myocardial hypertrophy is associated with an increase in coronary vascular resistance and a significantly decreased capillary numerical and volume density [3, 4]. Cardioplegia impairs the release of endothelium-derived relaxing factors from the coronary endothelium and damages the ultrastructure of the coronary endothelium [5, 6]. Impaired endothelium-dependent relaxation in coronary arteries results in arterial spasm and severely reduced blood perfusion at the level of the myocardium. Moreover, cardioplegic arrest abolishes the squeezing effect of heart contraction on coronary microvasculature and further compromises coronary blood perfusion. Myocardial hypertrophy is also associated with the decrement in high-energy phosphate levels and the depressed ability of mitochondrial to create energy [7, 8], which results in some extent of myocardial stunning and apoptosis before surgery [9]. Myocardial stunning occurs during cardioplegic arrest and can be initiated by selective cardiac troponin I (cTnI) degradation [10]. The generation of free radicals and intracellular calcium overload in stunned myocardium has been demonstrated to contribute significantly to myocardial dysfunction [10]. Cardioplegia arrest induces apoptotic signal cascades in endothelial cells and cardiac myocytes in the human myocardium [11, 12]. Myocardial apoptosis may play a key role in persistent myocardial dysfunction after open-heart surgery.

Recently, beating heart valve surgery has emerged as an effective alternative method to prevent cardioplegia-associated detrimental effects. Preliminary clinical practice suggested that operative vision of beating heart valve surgery was equal to that of traditional valve surgery and technical accuracy was not compromised [13, 14]. Moreover, three-dimensional architecture of the beating hearts facilitated examination of aortic and mitral valve during surgery [15, 16]. Our previous study demonstrated that keeping hypertrophied hearts beating with empty ventricles improved myocardial fluid homeostasis relative to cardioplegic arrest [17–19].

Normothermic normokalemic beating perfusion (NNBP), keeping the heart empty and beating, resembles close-normal physiological situation, avoids the utilization of high-potassium cardioplegia, and maintains the squeezing effect of heart contraction on vessels. Thus, we hypothesized that NNBP had the advantage over normothermic hyperkalemic cardioplegia arrest (NHCA) in improving myocardial tissue perfusion, depressing cardiomyocyte apoptosis and myocardial stunning, and preserving heart contractile function.

## 2. Materials and Methods

### 2.1. Ethics Statement

This study was conducted under an approval by the Institutional Review Board and Animal Care Committee of National Research Council of Canada and Huazhong University of Science and Technology.

### 2.2. Pressure Overload-Induced Left Ventricular Hypertrophy

Eighteen 6- to 8-week-old piglets were intubated and mechanically ventilated. A left thoracotomy was performed in the third intercostal space. A polyethylene band (5 mm in width) was tightened around ascending aorta with a peak systolic pressure gradient of 10 mmHg across the narrowing. Left ventricular (LV) hypertrophy occurred progressively as aortic constriction remained fixed with normal body growth. In six sham operated piglets, the band was pulled through ascending aorta; thus, no aorta narrowing developed after surgery. The chests were then closed, and the pigs were allowed to recover for 58.6 ± 7.2 days.

### 2.3. Experimental Protocol

Immediately, 28.6 ± 2.4 and 58.6 ± 7.2 days after aortic banding, all pigs underwent cine MRI to monitor the magnitude of LV hypertrophy. At 58.6 ± 7.2 days after banding, median sternotomy was accomplished under general anesthesia, and then pigs were transferred to MR region next to surgical room. Cine MRI was performed to assess heart function before cardiopulmonary bypass (CPB).

The pigs with severe LV hypertrophy were divided into two groups (*n* = 6 per group). Pigs in group I underwent the NNBP, and hearts were maintained at a empty and beating condition during a 60-minute preservation period. Pigs in group II received the NHCA, and hearts were kept in a quiescent and arrested situation at a similar period of time. Thereafter, CPB with standard cannulations was initiated during NNBP or NHCA. Krebs-Henseleit (K-H) solution was mixed with the pig blood in 1 : 1 ratio. The mixture was used to perfuse isolated hearts. Krebs-Henseleit solution is widely accepted as a physiologic perfusion medium and has been used for many years for heart perfusion. The K-H solution contained 118 mmol/L NaCl, 1.2 mmol/L MgSO_4_, 0.5 mmol/L ethylenediaminetetraacetic acid, 11 mmol/L glucose, 25 mmol/L NaHCO_3_, 1.75 mmol/L CaCl_2_, and 0.625% bovine serum albumin. The potassium concentration of the mixture was 3.4 and 16 mmol/L for NNBP and NHCA, respectively. Near infrared spectroscopic imaging was performed with the bolus administration of indocyanine green (ICG) to assess myocardial perfusion. After 60-minute CPB, the pigs were weaned off CPB and were transferred to neighboring MR area. Cine MRI was likewise performed to evaluate post-CPB myocardial function.

At the end of experiment, LV myocardial tissues were obtained, snap frozen in liquid nitrogen, and stored in −80°C for TUNEL staining and western blotting studies.

### 2.4. Myocardial Preservation under Cardiopulmonary Bypass

The internal mammary artery was catheterized to monitor arterial pressure. After heparinization, the aorta was cannulated for aortic perfusion. The superior and inferior vena cava were cannulated for venous return. The LV was vented directly by a catheter inserted through the left atrial appendage. CPB was instituted by using a membranous oxygenator (Maxima Plus; Medtronic, Cardiopulmonary Division; Anaheim, CA) with a flow rate at 50 mL/kg/min. Pigs were placed on total CPB by snaring the vena cava. After stabilization, the ascending aorta was cross-clamped. A double-lumen aortic root cannula was inserted for antegrade delivery of perfusate and simultaneous measurement of infusion pressure. All infusions of myocardium were administered at the pressure of 40 to 50 mm Hg. The ascending aorta was cross-clamped for 60 minutes.

### 2.5. TUNEL Staining

The cryopreserved tissue sections were fixed in 4% paraformaldehyde. Then, tissue sections were stained by TUNEL with In Situ Cell Death Detection Kit (Roche Diagnostics, Indianapolis, IN) according to the manufacturer's instructions. After TUNEL, the sections were mounted with Prolong Gold antifade reagent with DAPI (Invitrogen, Carlsbad, CA). Ten photographs (magnification, ×200) of each tissue section were taken under a fluorescence microscope and analyzed by two independent researchers. The number of apoptotic myocytes was expressed as the number of TUNEL-positive cells per 1000 total nuclei. Normal myocardium from sham operated pigs was used as negative control. Positive control was obtained from normal myocardial tissue kept at room temperature for one hour.

### 2.6. Western Blot Studies

Protein samples from the experimental animal myocardial tissues were fractionated on 12.5% SDS-PAGE and transferred to polyvinylidene (PVD) membrane (Sigma-Aldrich, Saint Louis, MO) and then blocked for one hour at room temperature with 5% nonfat dry milk in PBS containing 0.1% Tween-20 (PBST). The membranes were incubated with a polyclonal anti- troponin I antibodies (Biodesign, Saco, ME) and anti-caspase-3 (Santa Cruz Biotechnology, Santa Cruz, CA) at room temperature for two hours. The membranes were washed with PBST and then incubated with secondary antibodies either a rabbit anti-mouse or mouse anti-goat IgG conjugated to horseradish peroxidase. Immune complexes were visualized with the enhanced chemiluminescence detection system (Pierce, Rockford, IL). Relative quantification of immunoreactive bands was performed by Quantity One imaging software (Bio-Rad Laboratories, Hercules, CA). Each modified product was quantified as a percentage of the total amount of protein.

### 2.7. Near Infrared Spectroscopic Imaging

An infrared-sensitive CCD-array camera was positioned overtop the chest cavity, so that the heart filled the field of view. Consecutive images were acquired at a single wavelength of 800 nm and total acquisition time for 120 sequential images was approximately 2 minutes. An ICG bolus (16 mg/10 mL) was injected through cardiac perfusate line after aorta cross clamp. The bolus administration was performed in the third images after onset of acquisition, allowing the achievement of at least three pre-ICG heart images. Three minutes after injection of ICG bolus, blood samples were taken and plasma was analyzed for ICG content spectrophotometrically at 800 nm. Following each trial, a uniform reflectance standard (Kodak Gray Card, Eastman Kodak, Rochester, NY) was placed overtop the heart and a reference image sequence was acquired.

### 2.8. Cine MRI

Cine MRI was performed on a 3-Tesla MR scanner (Magneton Symphony Vision, Siemens AG, Erlangen, Germany). A phased array four-element body surface coil was utilized for signal reception. MR sequences were electrocardiography-triggered, breath hold, and acquired in oblique short-axis view. Cine images were acquired with a fast gradient echo sequence in oblique short-axis plane. The following parameters were used: field of view: 8 × 8 cm^2^, flip angle: 30 to 40°, echo time: 3.5 ms, repetition time: 9.2 ms, matrix: 256 × 256, and temporal resolution: 50 ms. For quantitative determination of morphology and function, 12–14 contiguous ventricular short-axis slices of 6 mm thickness were acquired from apex to base to cover the entire LV.

### 2.9. Cine MRI Analysis

Cine images data were analyzed by using freely available software (Segment, Version 1.8R0438, http://segment.heiberg.se). The epicardial and endocardial borders of the myocardium were manually traced on end-diastolic and end-systolic images at each anatomic level encompassing the entire LV. End-diastolic and end-systolic wall thickness were measured in six segments including the anteroseptal anterior, anterior-lateral, anterolateral, interior, and anteroseptal wall of LV in three contiguous midventricular slices. LV slice volumes were determined from end-diastolic and end-systolic images by multiplication of compartment area and slice thickness. Total LV volumes were calculated as the sum of all slices volumes. Stoke volume was calculated using the following equation: end-diastolic volume-end-systolic volume. Ejection fraction was determined as followed: end-diastolic volume-end-systolic volume/end-diastolic volume (%).

### 2.10. Near Infrared Spectroscopic Imaging Analysis

Image stacks were processed using programs developed in house, running under MATLAB (version. 5.3, MathWorks, Natick, MA). The resulting spectroscopic imaging was a two-dimensional (256 pixels × 256 pixels) image of absorbance at an ICG specific wavelength (800 nm). Each image was rationed to the reference image at 800 nm to provide absorbance data. Extraction of the absorbance values for a single pixel through the 120 images yielded the time course of relative ICG kinetics in specific region of hearts. Two regions of interest (ROIs) were located at the left anterior descending artery (LAD) region and myocardial tissue area, respectively. The comprehensive time courses of ICG absorbance were obtained at the two specific ROIs. Maximal absorbance was calculated from ICG time courses to evaluate myocardial blood flow.

### 2.11. Statistical Analysis

All data are presented as the mean ± standard deviation. Repeated measures analysis of variance was used to compare the ICG absorbance values varied with the passage of ICG. Differences among different regions at specific time points were isolated by Bonferroni* t*-tests. The percentage of TUNEL-positive myocytes, the percentage of cleaved caspase-3 or degraded cTnI, LV end-diastolic volume, LV end-systolic volume, ejection fraction, and stroke volume were compared with a paired* t*-test. Values of *P* < 0.05 were deemed significant.

## 3. Results

### 3.1. Ascending Aortic Diameters

**Figure 1:**
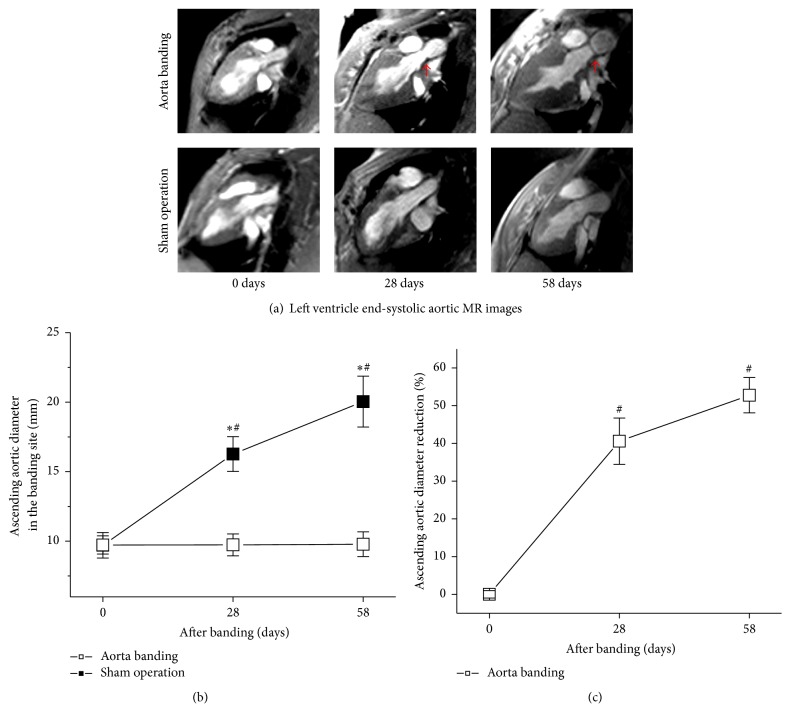
The aortic stenosis produced by banding the ascending aorta. (a) Aortic narrowing was manifest in aorta banding animals (red arrowheads). (b) Ascending aorta diameter in banding site remained unchanged, whereas animal growth was associated with a progressively increased diameter of the ascending aorta. (c) There were significant reductions in luminal diameter at average 28 and 58 days after aorta banding. Banding the ascending aorta caused a significant aortic luminal narrowing. ^*∗*^*P* < 0.05 versus sham operation. ^#^*P* < 0.05 versus 28 or 58 days.

The representative end-systolic aorta MR images immediately, average 28 and 58 days after ascending aortic banding, are illustrated in Figure 1(a). The ascending aortic stenoses were significant at average 28 and 58 days after banding (red arrowheads, Figure 1(a)).

Ascending aortic diameter at the banding site remained unchanged. They were 9.7 ± 0.8, 9.7 ± 0.7, and 9.8 ± 0.6 mm immediately, average 28 and 58 days after aortic banding, respectively (Figure 1(b)). Ascending aortic diameter progressively increased with the animal growth in sham operation pigs. They were 9.7 ± 0.9, 16.2 ± 1.2, and 20.0 ± 1.8 mm immediately, average 28 and 58 days after aortic banding, respectively (Figure 1(b)). Correspondingly, ascending aortic diameter was reduced by 40.5% ± 0.6% and 52.7% ± 0.4% at average 28 and 58 days after aortic banding (Figure 1(c)). These indicated that approximately eight-week aorta banding caused the significant ascending aortic narrowing.

### 3.2. LV Hypertrophy

**Figure 2:**
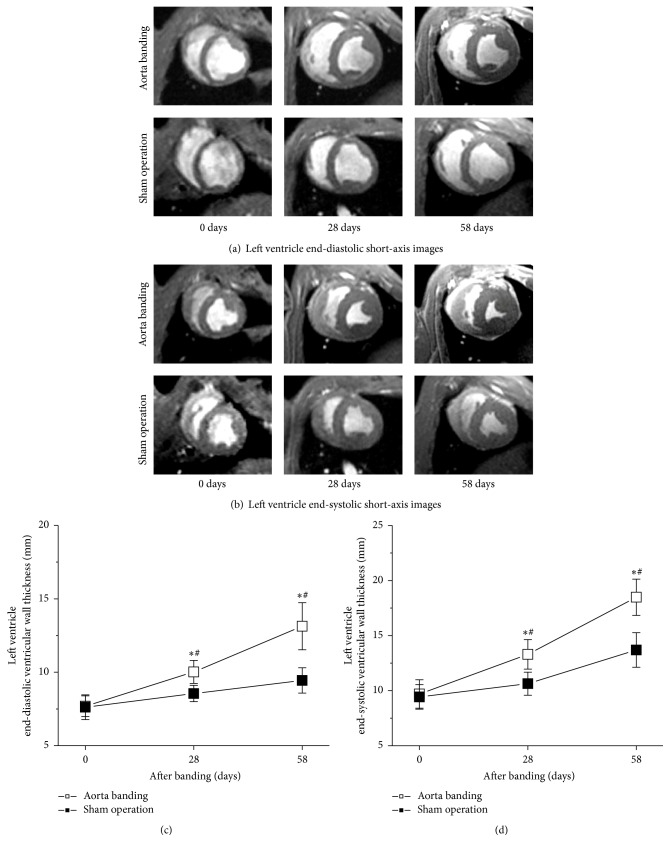
LV wall hypertrophy originated from aorta banding. (a-b) LV of aorta banding animals had thicker wall in comparison with sham operation animals. (c-d) LV end-diastolic and end-systolic wall thicknesses were substantially greater in aorta banding animals than in sham operation animals. The approximately eight-week banding period was sufficient to induce LV wall hypertrophy. ^*∗*^*P* < 0.05 versus sham operation. ^#^*P* < 0.05 versus 28 or 58 days.

LV end-diastolic and end-systolic short-axis images immediately, average 28 and 58 days after ascending aortic banding, are illustrated in Figures 2(a) and 2(b), respectively.

LV end-diastolic wall thickness was significantly greater in aorta banding than in sham operation (10.0 ± 0.7 versus 8.5 ± 0.5 mm at average 28 days after banding, 13.1 ± 1.6 versus 9.4 ± 0.8 mm at average 58 days after banding) (Figure 2(c)). Likewise, aorta banding displayed a markedly increased LV end-systolic wall thickness compared with sham operation (13.2 ± 1.3 versus 10.6 ± 1.0 mm at average 28 days after banding, 18.4 ± 1.6 versus 13.6 ± 1.5 mm at average 58 days after banding) (Figure 2(d)). These data were indicative of a markedly increased LV wall thickness at approximately 8 weeks after aortic banding.

### 3.3. Myocardial Tissue Perfusion

**Figure 3:**
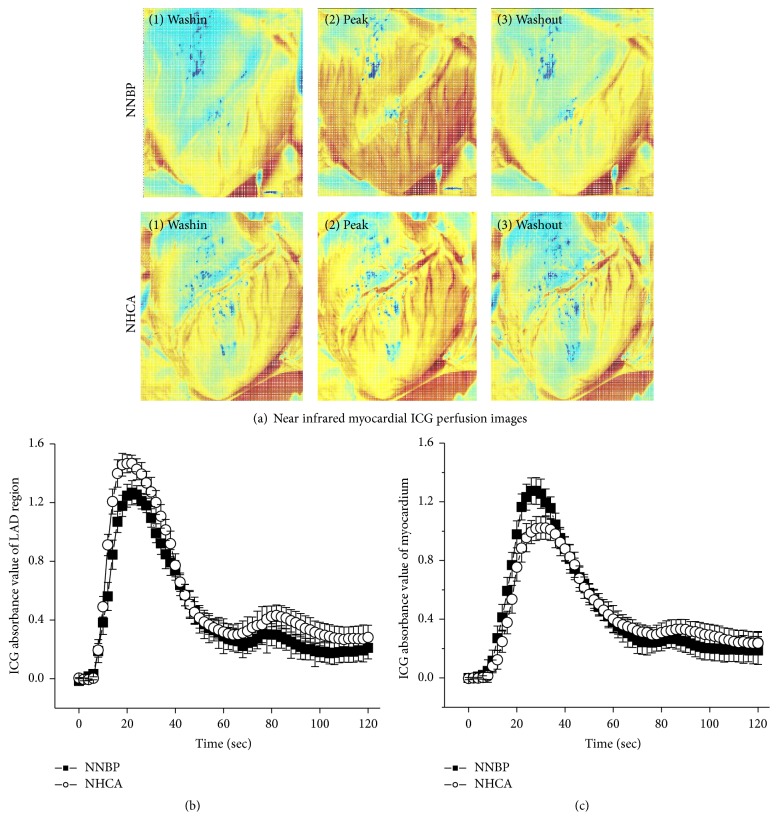
Near infrared myocardial ICG perfusion images and time-ICG absorbance value curves during NNBP and NHCA. (a) LV myocardium of NNBP became red more rapidly and intensively in comparison with NHCA. (b) ICG absorbance peak value of LAD region was moderately lower in NNBP than in NHCA. (c) ICG absorbance peak value of LV myocardium was significantly higher in NNBP than in NHCA. NNBP squeezed intracoronary blood flow more to enter myocardial capillaries relative to NHCA.

A bolus injection of ICG was associated with a quick appearance of red color, initially in the epicardial coronary arteries and then in myocardial tissue. Representative myocardial ICG perfusion images are illustrated in Figure 3(a). Red region of LV was bigger and more obvious in NNBP than in NHCA (Figure 3(a)).

Representative time courses for ICG passage through hearts are shown in Figures 3(b) and 3(c). The peak absorbance value of LAD region was moderately lower in NNBP (1.02 ± 0.07) than in NHCA (1.27 ± 0.09 a.u.) (Figure 3(b)). Conversely, the peak absorbance value of LV myocardium was significantly higher in NNBP (1.46 ± 0.06) than in NHCA (1.26 ± 0.08 a.u.) (Figure 3(c)). These suggested that cardiac contractile activity during NNBP squeezed intracoronary blood flow to distribute more into myocardial capillaries relative to cardioplegic arrest during NHCA.

### 3.4. Myocyte Apoptosis

**Figure 4:**
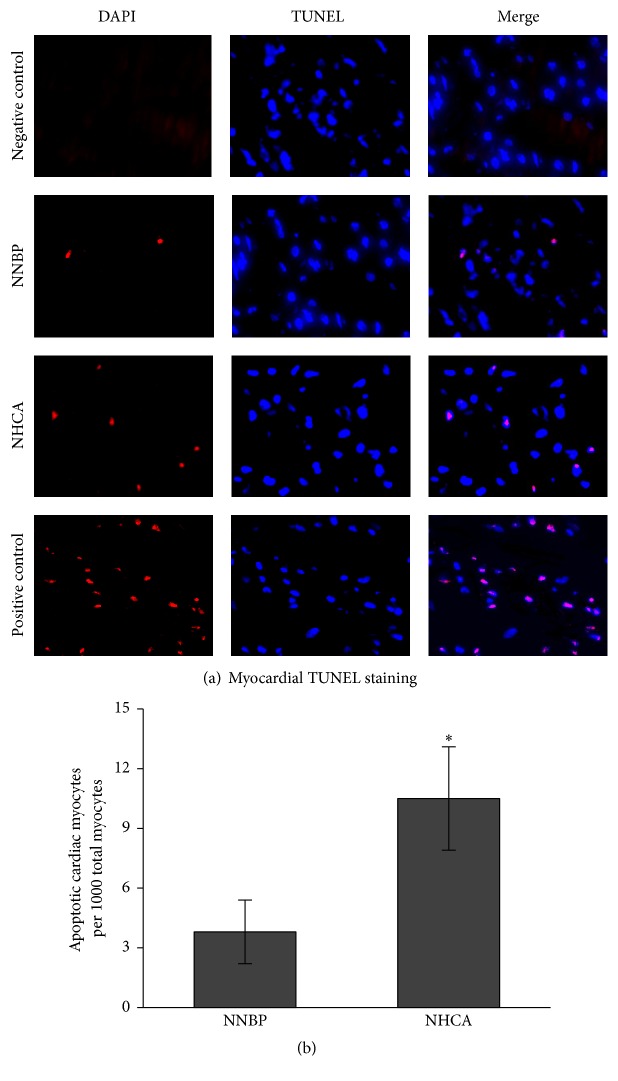
Myocardial TUNEL staining. (a) TUNEL-positive nuclei (red) were significantly less in NNBP compared with NHCA. (b) Apoptotic myocytes were statistically lower in NNBP than that in NHCA. ^*∗*^*P* < 0.05 versus NNBP.

TUNEL-positive nuclei (red) were more frequent in NHCA when compared with those in NNBP (Figure 4(a)). Percentage of TUNEL-positive myocytes in NNBP (3.8 ± 1.6/1000 nuclei) was statistically lower than that in NHCA (10.5 ± 2.6/1000 nuclei) (Figure 4(b)).

**Figure 5:**
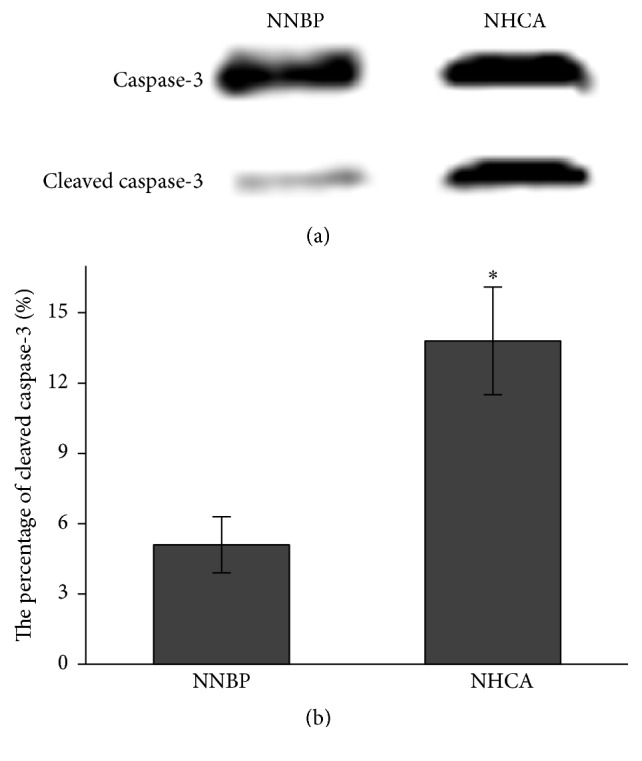
Western blot analysis of caspase-3 protein. (a) Cleaved caspase-3 was markedly decreased in NNBP as compared with that in NHCA. (b) The percentage of cleaved caspase-3 was significantly lower in NNBP than in NHCA. ^*∗*^*P* < 0.05 versus NNBP.

The cleavage of caspase-3 was consistently detected in pigs undergoing NNBP and NHCA. However, caspase-3 cleavage was more obvious in NHCA than in NNBP (Figure 5(a)). The percentage of cleaved caspase-3 was 5.1% ± 1.2% and 13.8% ± 2.3% in NNBP and NHCA, respectively (Figure 5(b)). These suggested that NNBP could inhibit cardiomyocyte apoptosis associated with NHCA.

### 3.5. Cardiac Troponin I Degradation

**Figure 6:**
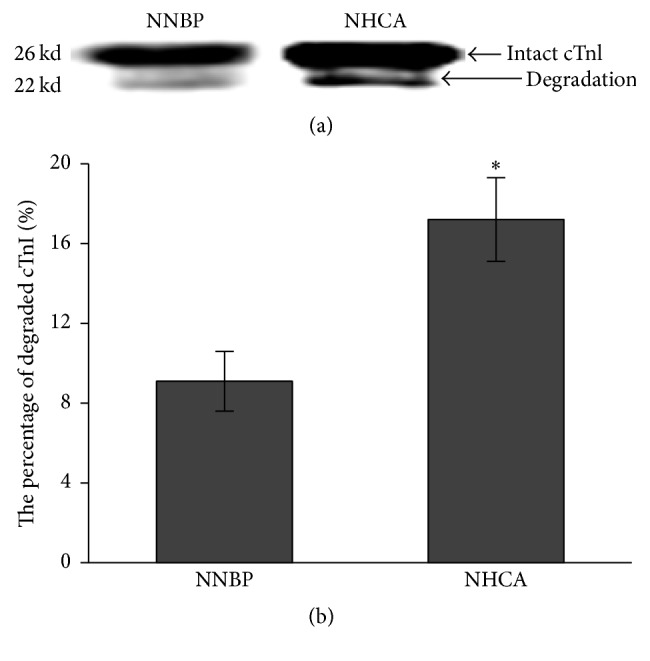
Western blot analysis of cTnI protein. (a) The intact 26-kd cTnI was partially degraded after CPB, as shown by the increasing quantity of 22-kd cTnI proteolysis product. (b) The percentage of degraded cTnI was significantly lower in NNBP than in NHCA. ^*∗*^*P* < 0.05 versus NNBP.

Intact and degraded cTnI were present in pigs undergoing NNBP or NHCA (Figure 6(a)). The specific degradation product of cTnI was thinner in NNBP when comparing with NHCA (Figure 6(a)). More importantly, the percentage of degraded cTnI was significantly lower in NNBP (9.1% ± 1.5%) than in NHCA (17.2% ± 2.1%) (Figure 6(b)). These implied that NNBP alleviated the degradation of cTnI associated with NHCA.

### 3.6. LV Contractile Function

**Figure 7:**
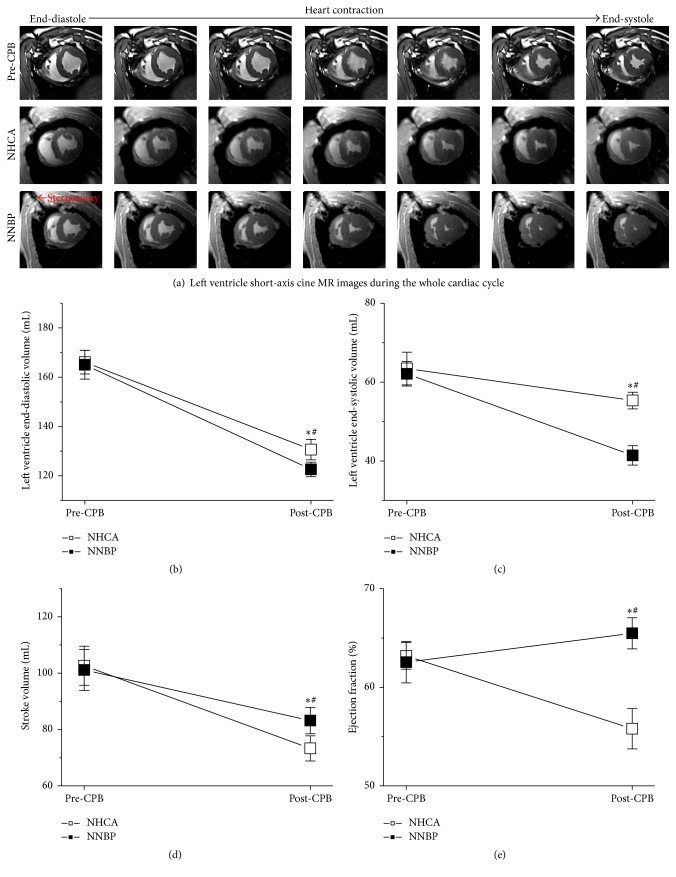
LV short-axis cine MR images and heart contractile function before and after NNBP or NHCA. (a) LV slice volume was moderately decreased in NNBP post-CPB when comparing NHCA post-CPB. (b-c) LV end-diastolic and end-systolic volumes were significantly lower in NNBP post-CPB than in NHCA post-CPB. (d-e) Stroke volume and ejection fraction were substantially higher in NNBP post-CPB than in NHCA post-CPB. NNBP was superior to NHCA in promoting cardiac contractile function recovery after CPB. ^*∗*^*P* < 0.05 versus NNBP or NHCA. ^#^*P* < 0.05 versus pre-CPB.

Representative short-axis MR cine images from end-diastole to end-systole during the whole cardiac cycle in pre-CPB, NHCA, and NNBP post-CPB are illustrated in Figure 7(a). NNBP post-CPB displayed a smaller LV slice volume in comparison with NHCA post-CPB (Figure 7(a)).

LV end-diastolic volume was significantly lower in NNBP post-CPB (122.5 ± 2.9) than in NHCA post-CPB (130.6 ± 4.1 mL) (Figure 7(b)). Moreover, LV end-systolic volume was markedly smaller in NNBP post-CPB (41.4 ± 2.4) than in NHCA post-CPB (55.3 ± 2.1 mL) (Figure 7(c)). Stroke volume was substantially higher in NNBP post-CPB (83.1 ± 4.6) than in NHCA post-CPB (73.3 ± 4.4 mL) (Figure 7(d)). Most meaningfully, the ejection fraction was higher in NNBP post-CPB (65.4% ± 1.5%) than in NHCA post-CPB (55.7% ± 2.0%) (Figure 7(e)). These demonstrated that NNBP improved the global LV contractile function relative to NHCA.

## 4. Discussion

The recent study was undertaken to examine the cardioprotective effect of NNBP on myocardial tissue perfusion, cardiac myocyte apoptosis, myocardial stunning, and heart contractile function in hypertrophied hearts. The major findings of this study are as follows: (1) the approximately eight-week aortic banding was an effective technique to induce pressure-overloaded myocardial hypertrophy; (2) NNBP enhanced myocardial tissue-level blood perfusion, inhibited cardiac myocyte apoptosis, decreased myocardial stunning, and improved LV contractile function in comparison with traditional NHCA.

Myocardial hypertrophy is associated with decreased capillary density, increased coronary arterial resistance, and comprised coronary vasodilator reserve [3, 4]. Abnormalities in coronary arterial circulation implies that hypertrophied hearts have less tolerance to reduction in coronary perfusion and are very sensitive to cardiac ischemia and reperfusion during cardioplegic arrest. Coronary endothelium regulates vasomotor tone and local tissue perfusion by producing endothelium-derived relaxing or contracting factors that act on vascular smooth muscle [20]. Hyperkalemic cardioplegia augments production of endothelium-derived contracting factor, impairs relaxation of the coronary endothelium, and increases vascular tone [5, 6]. Impaired coronary endothelium-dependent relaxation in coronary arteries might lead to coronary vasospasm, endothelial denudation, platelet adhesion, and aggregation [5, 6]. Consequently, cardioplegia compromises blood perfusion at the level of the myocardium especially in hypertrophied hearts. NNBP maintains normal electromechanical activity and avoids hyperkalemic cardioplegia, thus alleviating coronary endothelial dysfunction, preserving coronary endothelium integrity, and enhancing myocardial blood infusion. NNBP also strengthens myocardial tissue perfusion through its squeezing effect of myocardial contraction on coronary arterial and venous systems.

Several myocardial stresses during cardioplegic cardiac arrest, including ischemia-reperfusion injury, coronary endothelium dysfunction, neutrophil-mediated pathologic events, and oxygen-derived free radicals, have been reported to trigger cardiomyocyte apoptosis [11, 12, 21, 22]. Activation of apoptosis signal cascades in endothelial cells and cardiac myocytes was observed in animal and human myocardium after traditional cardioplegic arrest [11, 12, 21, 22]. Cleavage and activation of caspase-3 are recognized downstream effectors of apoptotic cell death.

Cardiomyocyte apoptosis may be involved in irreversible myocardial tissue damage and persistent ventricular dysfunction after cardioplegia surgery. Myocardial stunning is characterized by reversible contractile dysfunction despite restoration of blood flow without myocyte necrosis. cTnI plays a crucial role in the calcium-dependent muscle contraction. The specific and selective proteolysis of cTnI has been proposed to be the key mechanism for myocardial stunning as a result of activation of calcium-dependent proteases during cardioplegia arrest [10]. Myocardial hypertrophy results in the significant decrease in myocardial high-energy phosphate levels and mitochondrial volume density [7, 8]. Thus, hypertrophied cardiomyocytes are more susceptible to ischemic myocardial apoptosis and stunning after open-heart surgery. NNBP improves myocardial tissue blood perfusion, provides adequate nutrients and oxygen, washes out all the metabolic waste, and impedes the occurrence of ischemia and reperfusion injury. This study suggested that, in conditions of NNHP without cardioplegic cardiac arrest, the amount of apoptotic myocytes was reduced, the cleavage of caspase-3 and the cTnI degradation were inhibited, and the LV contractile function was improved in hypertrophied heart after open-heart surgery.

The occurrence of cardiomyocyte apoptosis increases with exposure to prolonged ischemia during cardioplegic arrest [22, 23]. Cardiomyocyte apoptosis plays a critical role in tissue damage and ventricular dysfunction after cardioplegic arrest [22, 23]. Cardioplegia has been reported to trigger myocardial stunning (reversible contractile dysfunction in the absence of cell death) [24]. Incomplete perfusion to all regions of heart is the important mechanism for cardiomyocyte apoptosis and myocardial stunning during open-heart surgery [21]. The recent study indicated that NNBP enhanced myocardial perfusion, depressed cardiac myocyte apoptosis, decreased myocardial stunning, and improved heart contractile function in comparison with traditional NHCA. We believed that improvements in myocardial perfusion are the fundamental mechanisms supporting the superiority of NNBP on myocardial apoptosis, stunning, and dysfunction.

There are several limitations to our study. We did not perform the histological examination of myocardial hypertrophy to clarify the intracellular collagen deposition and the hypertrophied cardiomyocytes. However, MRI data have indicated the significantly increased wall thickness. Long-term supracoronary banding of the ascending aorta was utilized to create myocardial hypertrophy. Pathological changes of hypertrophied pigs might be somewhat different from these of the patient hearts. Therefore, the recent findings could not be directly extrapolated into the clinical scenario. Another limitation of the study was that the study did not investigate the beneficial effect of empty-beating on the morphological integrity of coronary endothelium, the apoptosis occurring in endothelial cells, and the patency of microvasculature. Further studies are required to prove whether empty-beating technique prevents coronary endothelial dysfunction following cardioplegic arrest.

## 5. Conclusions

NNBP was superior to NHCA in enhancing myocardial tissue perfusion, inhibiting myocardial cTnI degradation, and alleviating cardiac myocyte apoptosis and preserving heart contractile function in hypertrophied hearts for valve surgery.
