# Corrigendum to “Preservation of Myocardial Perfusion and Function by Keeping Hypertrophied Heart Empty and Beating for Valve Surgery: An In Vivo MR Study of Pig Hearts”

**DOI:** 10.1155/2017/6490349

**Published:** 2017-12-31

**Authors:** Jian Wang, Bo Xiang, Jixian Deng, Hung-Yu Lin, Darren H. Freed, Rakesh C. Arora, Ganghong Tian

**Affiliations:** ^1^Department of Vascular Surgery, Union Hospital, Tongji Medical College, Huazhong University of Science and Technology, Wuhan 430022, China; ^2^National Research Council of Canada, 435 Ellice Avenue, Winnipeg, MB, Canada R3B 1Y6; ^3^Department of Physiology, Faculty of Medicine, University of Manitoba, 727 McDermot Avenue, Winnipeg, MB, Canada R3E 3P5; ^4^Department of Pharmacology and Therapeutics, Faculty of Medicine, University of Manitoba, 753 McDermot Avenue, Winnipeg, MB, Canada R3E 0T6; ^5^Division of Cardiac Surgery, University of Alberta Hospital, 8440-112 Street, Edmonton, AB, Canada T6G 2B7; ^6^Cardiac Science Program, Institute of Cardiovascular Science, St. Boniface General Hospital, 409 Tache Avenue, Winnipeg, MB, Canada R2H 2A6

In the article titled “Preservation of Myocardial Perfusion and Function by Keeping Hypertrophied Heart Empty and Beating for Valve Surgery: An In Vivo MR Study of Pig Hearts” [1], affiliation number one was given incorrectly. The corrected affiliation is shown above.

## References

[1] Wang J., Xiang B., Deng J. (2017). Preservation of myocardial perfusion and function by keeping hypertrophied heart empty and beating for valve surgery: an in vivo MR study of pig hearts. *BioMed Research International*.

